# Initial Displacement and Stress Distribution of Upper Central Incisor Extrusion with Clear Aligners and Various Shapes of Composite Attachments Using the Finite Element Method

**DOI:** 10.3390/dj10060114

**Published:** 2022-06-20

**Authors:** Pratchawin Laohachaiaroon, Bancha Samruajbenjakun, Ekachai Chaichanasiri

**Affiliations:** 1 Orthodontic Section, Department of Preventive Dentistry, Faculty of Dentistry, Prince of Songkla University, Hat Yai, Songkhla 90110, Thailand; pratchawin@gmail.com; 2 Department of Mechanical Engineering, Faculty of Engineering, Mahidol University, Phuttamonthon, Nakhon Pathom 73170, Thailand; ekachai.cha@mahidol.ac.th

**Keywords:** finite element method, orthodontics, composite attachment, clear aligners

## Abstract

A clear aligner is an esthetic and more comfortable option for patients who need orthodontic treatment. However, some types of tooth movement, such as extrusion, are difficult with this tool. Therefore, composite attachments have been suggested to improve tooth movement. This study aims to evaluate the initial displacement and stress distribution during upper central incisor extrusion using the conventional composite attachments. Maxillary models with the upper teeth, clear aligners, and composite attachments placed on the labial surface of the upper right central incisor were constructed. Four models were created to simulate upper central incisor extrusion: (1) without any composite attachment; (2) rectangular beveled attachment; (3) ellipsoid attachment; and (4) horizontal rectangular attachment. Clear aligners were designed to perform upper central incisor extrusion. The constructed models were analyzed using the finite element method. Initial displacement and stress distribution were analyzed. Output analysis found that the upper right central incisor in the model with a horizontal rectangular attachment had the greatest extrusive movement, followed by the model with ellipsoid attachment and the model with beveled attachment. Maximum compressive stress was seen at the cervical region of the composite attachment. Composite attachments including horizontal rectangular attachment, ellipsoid attachment, and rectangular beveled attachment can be used to perform upper central incisor extrusion.

## 1. Introduction

Nowadays, the number of adult patients who seek orthodontic treatment has increased [1], while the esthetic value of wearing an orthodontic appliance is a consideration. Therefore, new types of orthodontic appliances have improved appearance and comfort. One of them is the clear aligner, which is an esthetic and more comfortable option for patients who need orthodontic treatment due to its transparence, removability, flatness, and less thickness compared to the fixed appliance. Therefore, it has become popular [2].

Clear aligners sequentially move the teeth to the desired position by a pushing force created from the different shapes of aligners and teeth. However, some types of tooth movement, such as incisor extrusion, is challenging with the clear aligner due to the lack of a pushing surface. Previous studies found that incisor extrusion with clear aligners has the least amount of accuracy with an average of 30% compared to other types of tooth movement. Extrusion of upper central incisors appeared to be the least accurate of all (18.3%), followed by mandibular incisors (24.5%). Therefore, composite attachments have been suggested to improve tooth movement [3,4,5,6,7].

Composite attachments are small auxiliary composite buttons with a defined geometry that are attached to the surface of the teeth to improve both the retention of clear aligners and transmit force from clear aligners to the teeth [8]. According to the Invisalign^®^ system, the common shapes of attachments, also called conventional attachments, are rectangular, rectangular beveled, and ellipsoid [9]. Although attachments have great potential in tooth movement, knowledge for the proper selection in dental practice is limited due to few studies regarding their biomechanical properties [10]. A study by Savignano et al. [11] concluded that without any attachment, extrusion of an upper central incisor is impossible. The study also reported that the position of composite attachments showed a stronger influence on tooth movement, while different composite attachment shapes in the same position generated an equal extrusive force. However, a study by Costa et al. [12] used custom designed composite attachments that were modified from the conventional attachments to extrude the upper central incisor. They found that different attachment designs generated significantly different directions and amounts of forces. In both studies, the authors focused only on the extruded tooth, while the adjacent teeth were not considered at all. This conflict of biomechanical effects leads to a significant gap of information about the different biomechanical performances of the three original conventional attachments that are widely used in commercial clear aligners. However, the study of orthodontic biomechanics can be quite challenging. An effective way to obtain biomechanical data for orthodontic treatment is the finite element method (FEM) [13].

The FEM is a computer-based numerical method used to simulate complex geometrical objects and their physical properties. The FEM is a non-invasive and accurate method that provides useful data for an orthodontist to understand the biomechanical and physiological responses that occur in tissues such as the periodontal ligament and the alveolar bone [14]. Previous FEM studies on clear aligners found that composite attachments played an important role in precise tooth movement with a clear aligner [15]. In addition, studies also found that the Optimized Root Control Attachments^®^ from Invisalign^®^ can bodily move the upper canine distally [16] and bodily move the upper central incisors to close a diastema [15]. However, to our knowledge no study has evaluated the biomechanics of the different original designs of conventional attachments for upper incisor extrusion that demonstrate the least accurate movement with clear aligners. Therefore, this study aims to evaluate the initial displacement and stress distribution generated during central incisor extrusion with clear aligners and various shapes of composite attachments using the FEM.

## 2. Materials and Methods

**Figure 1:**
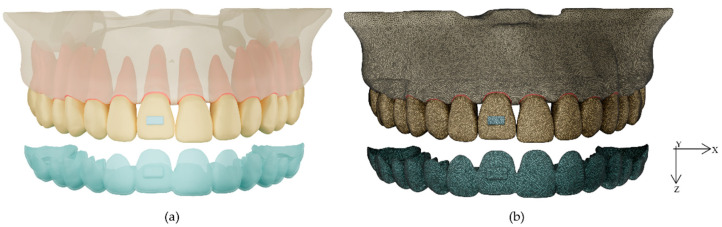
Maxilla model with PDL, upper teeth, clear aligner, and composite attachment: (**a**) 3D model and (**b**) finite element meshing model.

This study was approved by the ethics committee of the Faculty of Dentistry, Prince of Songkla University, Thailand (EC6106-022). A 3D geometric model was prepared of a maxilla arch that included the maxilla, periodontal ligament (PDL), upper teeth with the upper right central incisor intruded, clear aligners, and composite attachments (Figure 1a). The maxilla and upper teeth were constructed from cone-beam computed tomography (CBCT) data of a patient who had an Angle Class I skeletal relationship with well-aligned teeth and normal tooth shape. The CBCT image was taken with a 3D Accuitomo 170 (J. Morita Mfg. Corp., Kyoto, Japan) using a FOV of 170 mm × 120 mm and voxel size 0.25 mm. The image was imported into ITK-SNAP software [17] to generate the 3D geometric model.

**Figure 2:**
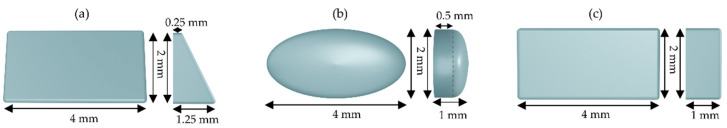
Dimensions of the (**a**) rectangular beveled attachment, (**b**) ellipsoid attachment, and (**c**) horizontal rectangular attachment [9].

The PDL was modeled on the root shape with a thickness of 0.25 mm [18]. Three shapes of composite attachments (Figure 2) were constructed on the upper right central incisor with the shape derived from the Invisalign^®^ system [9]. Specifically, the incisal margin of the composite attachments was placed 3.5 mm gingivally to the incisal edge. Both mesial and distal margins of the composite attachments were 2.5 mm away from the mesial and distal surfaces of the tooth, respectively. The clear aligners were made based on the target dentition to perform upper central incisor extrusion. Target dentition was developed by extrusion of the upper right central incisor and composite attachment with a 0.15 mm displacement along the tooth axis [16]. After that, the clear aligners were developed from an external offset of all teeth crowns and attachments at the target dentition. Clear aligner thickness was set at 0.5 mm [19]. Finally, four models with different shapes of composite attachments on the upper right central incisor were designed as follows:

Model 1: no composite attachment
Model 2: rectangular beveled attachment
Model 3: ellipsoid attachment
Model 4: horizontal rectangular attachment

The model components were converted to finite element solid meshes with 4-node tetrahedral elements using Patran software (MSC Software Corp., Newport Beach, CA, USA). All components were considered as linear elastic, isotropic, and homogeneous material. Material properties [16,20] and mesh size were set according to Table 1.

The meshing process produced a total of 1,467,263 nodes and 5,338,190 elements in a model without a composite attachment, 1,472,483 nodes and 5,355,482 elements in a model with a rectangular beveled attachment, 1,469,009 nodes and 5,342,773 elements in a model with an ellipsoid attachment, and 1,470,269 nodes and 5,346,694 elements in a model with a horizontal rectangular attachment (Figure 1b).

Bonded contacts were set at the interfaces between the bone and PDL, PDL and teeth, and teeth and composite attachment. Surface-to-surface contact was used between the clear aligner and teeth as well as the clear aligner and the composite attachment. Fixed supports were applied on the upper part of the maxilla. A friction coefficient of µ = 0.2 was used between the aligner and teeth as well as the aligner and the composite attachment [21].

All meshed components were imported into FEM software (Marc Mentat, MSC Software Corp., Newport Beach, CA, USA). To simulate of a clinical event, the clear aligner was inserted into the dentition using displacement control. After the clear aligner was seated into the dentition, all displacement control functions were removed to allow force delivery from the clear aligner to the teeth.

After the simulation was done, the results, including the initial displacement of teeth and stress distribution on teeth, composite attachments, and PDL, were analyzed. The initial displacement was evaluated by nodal displacement on the incisal edge, labial cemento-enamel junction (CEJ), and root apex.

## 3. Results

### 3.1. Initial Displacement

**Figure 3:**
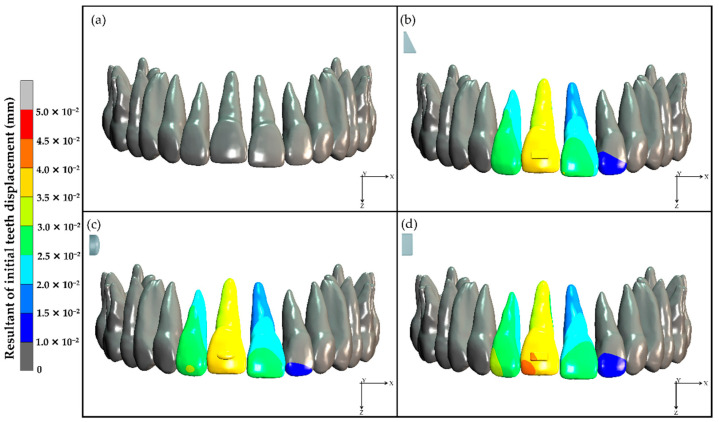
Resultant values of initial teeth displacement: (**a**) no composite attachment, (**b**) rectangular beveled attachment, (**c**) ellipsoid attachment, and (**d**) horizontal rectangular attachment.

The initial teeth displacements were recorded in 3-dimensions. The FEM study showed the resultant values of initial displacement of teeth at the upper right lateral incisor, upper right central incisor, and upper left central incisor in models each with a composite attachment (Figure 3).

#### 3.1.1. Upper Right Central Incisor

**Figure 4:**
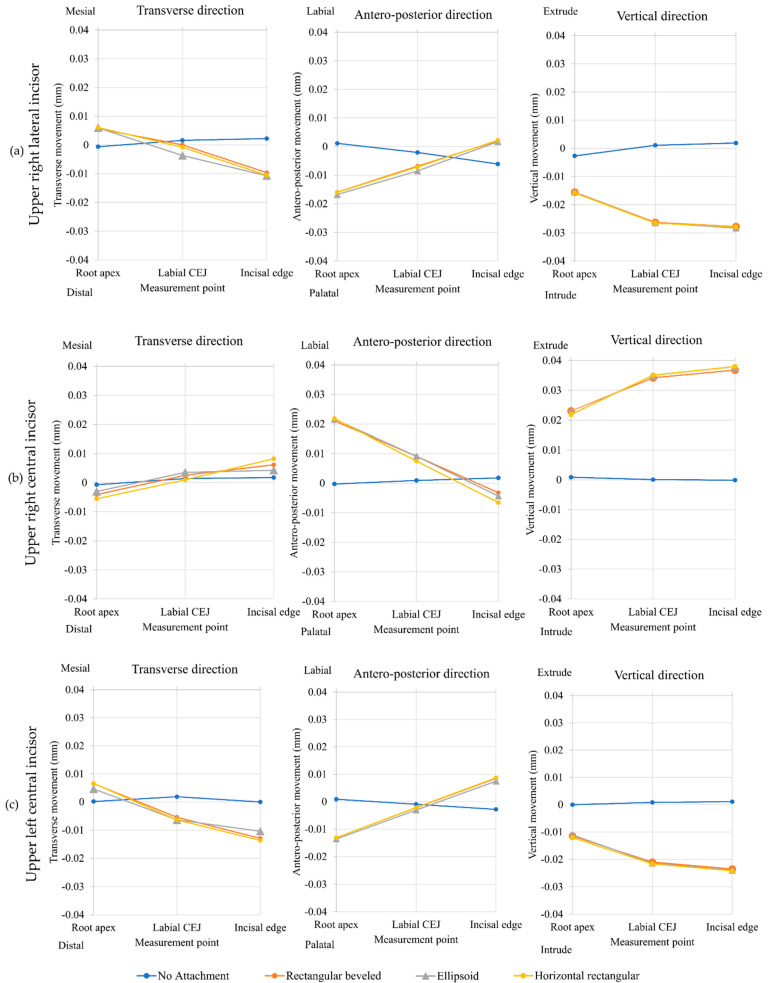
Nodal displacement results: (**a**) upper right lateral incisor, (**b**) upper right central incisor, and (**c**) upper left central incisor.

Initial displacement of the upper right central incisor is shown in Table 2 and Figure 4. Extrusive movement of the upper right central incisor was seen in the three models each with a composite attachment. When considering the incisal edge, the model with the horizontal rectangular attachment had the greatest extrusive movement (0.037991 mm), followed by the model with the ellipsoid attachment (0.037606 mm) and the model with the rectangular beveled attachment (0.036786 mm). The model without composite attachment demonstrated little intrusive movement (0.000105 mm).

#### 3.1.2. Upper Right Lateral Incisor and Upper Left Central Incisor

Intrusion and proclination showed the same pattern for all models with the composite attachment, while the model without a composite attachment showed negligible initial displacement of teeth (Figure 4).

### 3.2. Stress Distribution

#### 3.2.1. Stress Distribution in Tooth and Composite Attachment

**Figure 5:**
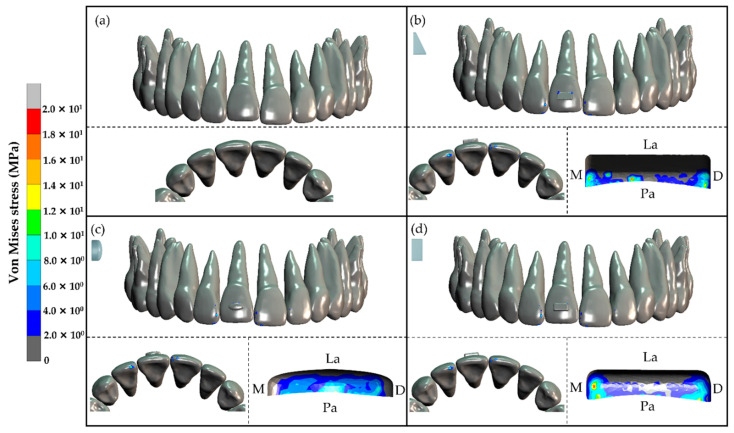
Distribution of von Mises stress on the upper anterior teeth in the frontal view, incisal view, and enlarged cervical view of the composite attachments: (**a**) no composite attachment, (**b**) rectangular beveled attachment, (**c**) ellipsoid attachment, and (**d**) horizontal rectangular attachment. M, Mesial; D, Distal; La, Labial; Pa, Palatal.

In the three models with composite attachment, the von Mises stresses were moderate to high at the cervical region of the composite attachment attached on the upper right central incisor. However, low stress was found at the crown of the upper right central incisor. Moreover, high stress was also found at the incisal edge and mesial surface of the upper right lateral incisor and upper left central incisor. The model without composite attachment had very low stress at all areas (Figure 5).

#### 3.2.2. Stress Distribution in the PDL

**Figure 6:**
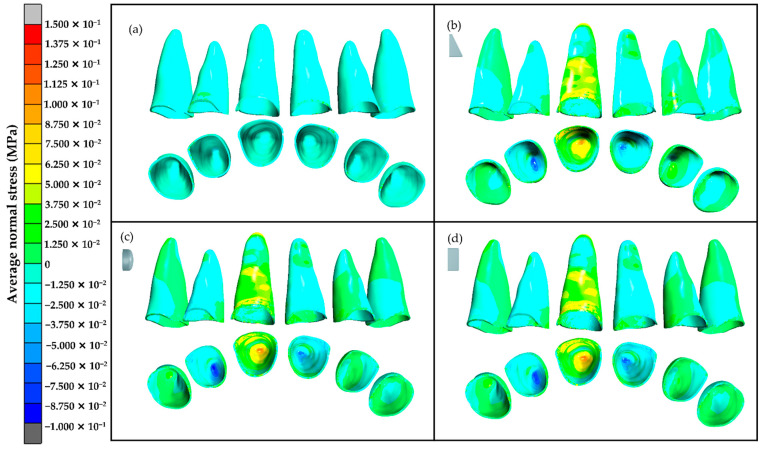
Distribution of the average normal stress at the upper anterior teeth PDL: (**a**) no composite attachment, (**b**) rectangular beveled attachment, (**c**) ellipsoid attachment, and (**d**) horizontal rectangular attachment.

In this study, the average normal stress, which is equal to the average of normal stress in the x, y, and z axes (*σ_x_*, *σ_y_*, and *σ_z_*), was used to study PDL stress distribution [20]. The average normal stress results showed that in the three models each with a composite attachment, the maximum tensile stress in the PDL was found at the apical area of the upper right central incisor that ranged from 0.125 MPa to 0.130 MPa. Additionally, maximum compressive stresses were seen at the apical areas of the upper right lateral incisor and upper left central incisor that ranged from 0.073 MPa to 0.090 MPa (Table 3 and Figure 6).

## 4. Discussion

The FEM is widely used in the orthodontic field to analyze orthodontic appliances, stress strain distribution in the periodontium, simulation of orthodontic tooth movement, and clear aligner biomechanics [14]. It has been demonstrated that the results from FEM provide acceptable accuracy to predict a clinical outcome [22].

This current FEM study evaluated the upper central incisor extrusion mechanics of a clear aligner. In the model without a composite attachment, the clear aligner was unable to extrude the right upper central incisor. This was consistent with a previous clinical study from Kravitz et al. [3] who found that the upper central incisor showed the least accurate tooth movement with a clear aligner (18.3% accuracy). This was due to the anatomy of the upper central incisor that did not have enough undercut, which caused the aligner to have a poor grasp of the tooth during a vertical pull. Therefore, prescribing even minor extrusive movements might require a composite attachment.

Upper right central incisor extrusion was seen in the models with a horizontal rectangular attachment, ellipsoid attachment, and rectangular beveled attachment. In addition, not only did the upper right central incisor extrude but also palatally tipped even though the clear aligners were designed to extrude the upper central incisor along the tooth axis. This occurred because of extrusion mechanics by the composite attachment and clear aligner. The extrusive force from the clear aligner was applied at the cervical portion of the composite attachment on the upper right central incisor, which could be seen from the von Mises stress results. When the extrusive force was applied to the cervical portion of the composite attachment, which was located anteriorly to the center of resistance of the upper central incisor, the force vector went through the front of the center of resistance and created a palatal crown tipping moment. This moment caused the crown of the upper right central incisor to tip palatally while the root apex tipped labially.

According to the initial displacement results, we found that the three shapes of composite attachments (horizontal rectangular, ellipsoid, and rectangular beveled) were essential to help extrude the upper central incisor. A composite attachment provides a grip for the clear aligner to grab the upper central incisor and transfer the force to extrude it. The results of the composite attachments of this current study were in line with previous clear aligner studies using the FEM [15,16,19]. In other words, composite attachments were shown to be necessary to improve the accuracy of tooth movement in various tooth movement types in upper canine retraction [16], upper anterior diastema closure [15], and lower premolar rotation [19]. However, those studies did not compare the effects of different shapes of composite attachments.

All models with a composite attachment resulted in effective initial tooth extrusion displacement of the upper right central incisor. The model with the horizontal rectangular attachment produced the greatest tooth extrusion (0.037991 mm), which was greater than the model with the ellipsoid attachment 0.000385 mm and greater than the model with the rectangular beveled attachment 0.001205 mm. However, these differences were very small and were not clinically significant. This occurred because the three composite attachment shapes in this study also had a surface that was tangent to the extrusive force to receive the force from the clear aligner. This corresponded to the stress distribution results that showed von Mises stresses at the cervical region of all composite attachments.

The upper right lateral incisor and upper left central incisor served as the anchorage teeth for the upper right central incisor extrusion. These anchorage teeth showed a von Mises stress distribution on the incisal edge causing them to intrude and procline. The proclination came from the intrusive force that passed anteriorly to the center of resistance that created a proclination moment on both teeth.

Because of the teeth displacement, the teeth transferred the stress to the PDL. The stress distribution results showed the same pattern for all models with a composite attachment. When the upper right central incisor was extruded, it caused tensile stress at the apical area of the PDL. The maximum tensile stress was 0.130 MPa in the horizontal rectangular model. However, the maximum compressive stress of 0.090 MPa was found at the apical area of the upper right lateral incisor in the same model. The compressive stress at the apical area of the upper right lateral incisor and upper left central incisor ranged from 0.073 MPa to 0.090 MPa, which was greater than the capillary blood vessel pressure of 0.0047 MPa according to Schwarz’s optimal force concept [23,24]. Therefore, the properties of the clear aligner material and a movement of 0.15 mm per aligner stage of extrusion, as mentioned earlier in the materials and methods section, may cause intrusive forces in the anchorage teeth that are greater than Schwarz’s optimal force level.

Although this FEM study provided biomechanical information of a clear aligner with various shapes of composite attachments to extrude an upper central incisor, the data included only the initial movement when the teeth started to move into the PDL space. Furthermore, a future study should be done to include other variables, such as various sizes and positions of the composite attachments to determine the different biomechanical effects.

## 5. Conclusions

This study demonstrated the initial extrusion displacement of the upper right central incisor with clear aligners and various shapes of composite attachments using the FEM.

1. The three composite attachment shapes including horizontal rectangular attachment, ellipsoid attachment, and rectangular beveled attachment can be used to perform upper central incisor extrusion.
2. Upper central incisor extrusion was caused by a force from the clear aligner applied at the cervical portion of the composite attachment.
3. In the three models each with a composite attachment, the stress distribution in the PDL was found to have the same pattern. Maximum tensile stress was found around the apical area of the upper right central incisor. Maximum compressive stresses were observed around the apical area of the upper right lateral incisor and left central incisor since these teeth served as the anchorage for the upper right central incisor extrusion.

## Figures and Tables

**Table 1:** Material properties and mesh size.

Components	Young’s Modulus (MPa)	Poisson’s Ratio	Mesh Size (mm)
Maxilla	1.37 × 10^3^	0.30	0.2–0.5
PDL	6.67 × 10^−1^	0.45	0.1
Teeth	1.96 × 10^4^	0.30	0.2
Composite attachment	1.25 × 10^4^	0.36	0.2
Clear aligner	528	0.36	0.2

**Table 2:** Nodal displacement of the upper right central incisor. Transverse direction (x) represents mesial (+) and distal (−) movement. Antero-posterior direction (y) represents labial (+) and palatal (−) movement. Vertical direction (z) represents extrusion (+) and intrusion (−) movement.

Models	Location	Displacement (×10^−2^ mm)		
		Transverse (x)	Antero-Posterior (y)	Vertical (z)
No composite attachment	Root apex	−0.0680	−0.0265	0.0886
	Labial CEJ	0.1450	0.0944	0.0078
	Incisal edge	0.1789	0.1796	−0.0105
Rectangular beveled attachment	Root apex	−0.4063	2.1039	2.3086
	Labial CEJ	0.2583	0.9132	3.4200
	Incisal edge	0.6166	−0.3218	3.6786
Ellipsoid attachment	Root apex	−0.3020	2.1653	2.3101
	Labial CEJ	0.0356	0.9139	3.4731
	Incisal edge	0.4274	−0.4352	3.7606
Horizontal rectangular attachment	Root apex	−0.5532	2.1848	2.1896
	Labial CEJ	0.5533	0.7496	3.5094
	Incisal edge	0.8252	−0.6546	3.7991

**Table 3:** Maximum of average normal stress at PDL around root apex: tensile stress as positive (+) values and compressive stress as negative (−) values.

Models	Teeth	Maximum Average Normal Stress at PDL around Root Apex (MPa)
No composite attachment	URLI	<0.001
	URCI	<0.001
	ULCI	<0.001
Rectangular beveled attachment	URLI	−0.076
	URCI	0.125
	ULCI	−0.073
Ellipsoid attachment	URLI	−0.090
	URCI	0.128
	ULCI	−0.073
Horizontal rectangular attachment	URLI	−0.090
	URCI	0.130
	ULCI	−0.081

URLI, upper right lateral incisor; URCI, upper right central incisor; ULCI, upper left central incisor.

## Data Availability

Not applicable.
